# Down with Retirement: Implications of Embodied Cognition for Healthy Aging

**DOI:** 10.3389/fpsyg.2016.01184

**Published:** 2016-08-09

**Authors:** Bernhard Hommel, Armin Kibele

**Affiliations:** ^1^Cognitive Psychology Unit and Leiden Institute for Brain and Cognition, Leiden University, LeidenNetherlands; ^2^Institute for Sports and Sport Science, University of Kassel, KasselGermany

**Keywords:** aging, aging and longetivity, cognitive enhancement, retirement, loneliness, dopamine, body strength, training

## Abstract

Cognitive and neurocognitive approaches to human healthy aging attribute age-related decline to the biologically caused loss of cognitive-control functions. However, an embodied-cognition approach to aging implies a more interactive view according to which cognitive control emerges from, and relies on a person’s active encounters with his or her physical and social environment. We argue that the availability of cognitive-control resources does not only rely on biological processes but also on the degree of active maintenance, that is, on the systematic use of the available control resources. Unfortunately, there is evidence that the degree of actual use might systematically underestimate resource availability, which implies that elderly individuals do not fully exploit their cognitive potential. We discuss evidence for this possibility from three aging-related issues: the reduction of dopaminergic supply, loneliness, and the loss of body strength. All three phenomena point to a downward spiral, in which losses of cognitive-control resources do not only directly impair performance but also more indirectly discourage individuals from making use of them, which in turn suggests underuse and a lack of maintenance—leading to further loss. On the positive side, the possibility of underuse points to not yet fully exploited reservoirs of cognitive control, which calls for more systematic theorizing and experimentation on how cognitive control can be enhanced, as well as for reconsiderations of societal practices that are likely to undermine the active maintenance of control resources—such as retirement laws.

Medical, societal, and economic progress has led to a rather dramatic increase of longevity and population aging in Western societies. Even so-called “healthy aging” often comes with noticeable decline in psychological and physiological functions, however. Particularly problematic is the increasing impairment of cognitive-control functions underlying goal-directed planning, impulse control, working memory, and related processes, which goes hand-in-hand with the loss of the underlying frontal and striatal dopaminergic supply (Umegaki et al., 2008; Cools and D’Esposito, 2011). Myriads of aging studies have concentrated on the targeted processes and the degree of impairment as a function of age(ing). As indicated by the three boxes of **Figure 1**, this approach is based on the idea that aging reduces the amount and/or quality of available cognitive-control resources, which in turn impairs the quality of the person’s actions and the resulting performance on control-heavy tasks, be it in everyday life or laboratory challenges designed to assess control abilities.

**FIGURE 1:**
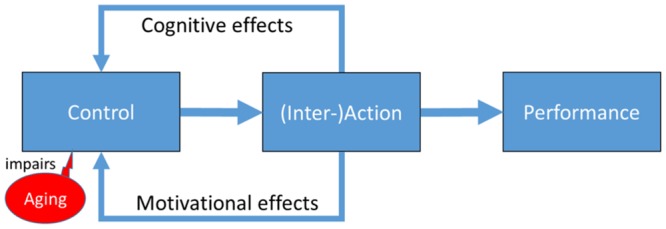
**Cognitive control as independent and dependent variable**.

While this approach seems to make perfect sense, it underestimates the complexity of the interactions between control processes and their application by failing to take the embodiment of cognitive control into consideration. The idea that human cognition is embodied relies on the assumption that cognitive processes and functions should not just be taken as a given, as the common information-processing approach to cognition suggests, but as abilities that emerge from active exchange with one’s physical and social environment (Wilson, 2002; Hommel, 2015, 2016). Among other things, this perspective draws attention to the question how people deal with the emergence or disappearance of particular abilities, and what they (could) do to compensate for cognitive and other impairments. Applied to cognitive aging, this suggests to consider natural/biological reductions of cognitive resources not as a given but as a change of the interaction between individual and environment. In other words, cognitive control resources should be considered as both an independent variable (that determines how well control challenges can be met) and a dependent variable (that is affected by dealing with these challenges).

In the present context, we focus on two processes that target cognitive control and that are likely to play a crucial role in cognitive aging. One of these processes is captured by the upper “cognitive effects” route in the figure. If cognitive control is mainly taken as a resource that is reduced in availability as age increases, all that an agent could do to maintain some level of quality of his or her actions and performance is to reduce the amount of required capacity—either by overlearning the action or by choosing less control-demanding actions. From an embodied perspective, however, the connection between cognitive control and action is not uni- but bidirectional. This means that actively exerting cognitive control implies the effective exercise of the underlying processes, which in turn promotes their maintenance. If so, control capacities would not need to be seen as fixed and exclusively regulated by biological factors but, rather, as depending on active use, not much different from a muscle. Even if this may not fully compensate for biological constraints it could very well reduce and dampen their impact.

The other process we focus on is captured by the lower “motivational effects” route in the figure. There is increasing evidence that self-representation and perceived agency relies on active control of one’s body and its effects on the environment (Hommel, 2015; Ma and Hommel, 2015). This in turn suggests that perceiving oneself as an agent need not be taken as a given and active agency as a consequence, but rather as emerging from one’s interactions with one’s environment. With respect to aging, this suggests that the reduced availability of cognitive control resources may not only have direct effects on the quality of actions and the resulting performance, but may also have indirect repercussions for the availability of cognitive control and/or the motivational forces necessary to make active use of them. Abandoning particular actions would lead to perceiving oneself as “someone not performing these kinds of actions”—as a (partial) non-agent that is. If we assume that the motivation to make active use of one’s control resources relies on one’s self-representation as an active agent, this implies that age-related inactivity can lead to the underuse of available control resources. Indeed, there is increasing evidence for substantial interactions between cognitive capacity and motivation in general and in aging in particular (for a comprehensive overview, see Braver et al., 2014), and increasing support for the possibility that substantial amounts of age-related performance deficits actually reflect motivational impairments (e.g., Ennis et al., 2013).

In the following, we will elaborate these considerations, and discuss the (often still preliminary) evidence for the existence of both cognitive and motivational effects on cognitive control, with respect to three important aging-related (and to some degree interrelated) phenomena.

## Aging and Dopaminergic Supply

Many cognitive processes show some degree of aging-related decline but cognitive-control processes, which orchestrate the more basic processes, are hit particularly hard. While it is difficult to tell cause from effect in this matter, this is likely to do with the particularly strong shrinkage of the frontal lobe, which houses many components of control networks, and the reduction of dopaminergic supply, which is fueling control structures and plays a key role in integrating control relevant cognitive and motivational processes and networks (Braver et al., 2014), with increasing age. Hence, there is ample evidence for aging-related reductions of control-specific resources.

But there is also evidence that engaging in cognitive control might compensate for at least some of these reductions. While tonic dopaminergic activity is assumed to energize exploratory behavior (Niv et al., 2006), engaging in exploratory behavior seems to increase phasic dopaminergic activity (Düzel et al., 2010)—a kind of self-maintaining loop. Unfortunately, the more elderly become aware of reduced cognitive resources, the more they avoid exploring situations that create uncertainty and surprise (de Bruin et al., 2010). This suggests that aging individuals may not fully exploit their potential to refill control resources through actively exposing themselves to as much uncertainty and surprise as possible—and thus reduce cognitive effects of control exercise.

In addition to such cognitive effects, motivational effects might also be involved. It has been argued that the motivation to expose oneself to new situations and environments may rely on the ability to anticipate and build predictive models based on hippocampal episodic memory (Düzel et al., 2010). However, extrapolating previous experience to create models of the future requires the availability of such experience, which in turn calls for the very activities that elderly seek to avoid with increasing age. The result is again a downward spiral that prevents elderly from making full use of their remaining cognitive potential.

## Aging and Loneliness

Feelings of social isolation and loneliness contribute significantly to the risk of elderly individuals to develop depressive symptoms and mental ill-health (Cacioppo et al., 2006), and reduced social interaction is associated with anxiety and decreases in cognitive functioning (Barnes et al., 2006). This has motivated numerous interventions to increase social interaction in older persons, unfortunately with little success so far (Dickens et al., 2011)–presumably for two reasons. First, aging is associated with reduced mobility and progressive mortality of friends and peers. This objectively provides elderly with fewer opportunities to expose themselves to social encounters and engage in social communication. Social interactions are prime examples for situations with a particularly high degree of uncertainty, which renders them ideal for practicing the very cognitive-control functions that are the most endangered in the aging individual. The natural loss of social networks with increasing age has thus particularly dramatic consequences for the elderly: while maintaining their cognitive-control functions would actually call for more interaction, they get less—an underuse of the cognitive training effect that social interactions provide.

There are also reasons to assume that motivational effects play a role. Fewer opportunities to engage in social interactions lead to a subjective loss of communicative abilities and coping capabilities, and to stronger feelings of insecurity and reduced feelings of safety (Gabriel and Bowling, 2004). This is likely to render social situations less and less rewarding (cf., Düzel et al., 2010), which will in turn make elderly individuals avoid social situations, presumably even more than justified by the actual loss of social skills. In any case, we see the same vicious circle: Objective losses of abilities and opportunities to practice control skills lead to the increased avoidance of situations in which such skills could be practiced and their impairment could be compensated for.

## Aging and Body Strength

Age-related loss of body strength has been reported in numerous studies (e.g., Mitchell et al., 2012). This loss is associated with impaired postural control and continuously increasing risk of falling (e.g., Granacher et al., 2008), which both can strongly limit the opportunities to engage in physical and social interactions. Losing body strength can impair performance in various ways. For one, it reduces the quality and accuracy of motor behavior, and by making the translation of action plans into overt behavior less predictable. For another, it increases the control demands of physical and social action, which together with the naturally decreasing control resources increasingly limits the action repertoire. Even actions as well-practiced as walking can become de-automatized and control-demanding (Li and Lindenberger, 2002), and often recruit more extended brain areas as age increases. This in turn puts increasingly high demands on the increasingly impaired cognitive-control processes, as indicated by observations that declines in postural control and risk of falling go hand in hand with decreases in cognitive functioning (e.g., Hsu et al., 2012; Best et al., 2016) and that performance on cognitive-control tasks predicts the risk of falling in the elderly (Buracchio et al., 2011).

As one would expect, strength training ha**s** the potential to maintain and enhance body strength in both young and older individuals, and training programs targeting strength, balance, and coordination can improve postural control and prevent falling (e.g., Barry and Carson, 2004; Granacher et al., 2011). However, more interesting for present purposes are findings that body-strength training improves cognitive functioning and conflict resolution (e.g., Liu-Ambrose and Donaldson, 2009; Liu-Ambrose et al., 2010). This suggests that supporting bodily functions to improve physical interactions with one’s environment can feed back to cognitive control and help maintaining its functioning—as the cognitive feedback loop of our model suggests.

Though admittedly still somewhat indirect, there is some evidence pointing to the possibility of a motivational feedback loop as well. In younger adults, posture has been demonstrated to affect emotional and motivational behavior and decision-making (Neumann et al., 2003), commonly in the sense that approach-associated postures lead to more positive emotions and evaluations than avoidance-associated postures do. More recent studies have revealed that posture also affects self-representation. For instance, at least individuals from Western societies have a greater sense of power when assuming expansive posters, such as standing upright and spreading out their hands or feet (e.g., Park et al., 2013). Importantly, if we consider how posture is affected by aging, it makes sense to expect that decreased posture control is associated with more negative emotions, more pessimistic judgments, and a reduced sense of power. Given that perceived self-efficacy is a key predictor of the likelihood to engage in body-related training at higher age (e.g., Schutzer and Graves, 2004; Costello et al., 2011), it makes sense to assume the existence of a motivational loop from interaction to cognitive control as well: perceiving oneself as being increasingly powerless and inefficient can prevent people from engaging in activities suited to overcome these deficits at least to some degree (cf., McAuley and Blissmer, 2010; Falck et al., 2016).

## Implications

We hope that these examples illustrate the interdependence of cognitive control and its active use during the aging process. Consideration of this mutual interdependence suggests to conceive of cognitive control not as an independent variable that determines the quality of control-demanding performance but, rather, as a skill that emerges from and relies on interactions with one’s physical and social environment. Accordingly, aging-related losses in control resources may not only reflect natural decline but also the lack of exploiting one’s potential to counteract this decline by making use of one’s remaining control abilities. Indeed, given that elderly individuals are biased toward affectively positive events (the positivity effect: Reed et al., 2014) and find cognitive effort more costly (Westbrook et al., 2013), it makes sense to assume that they are systematically motivated to under-use their cognitive capacity.

Before discussing the implications of this insight, we note that our arguments are rather conservative. Given that functional and neural descriptions of the same system are theoretically equivalent, finding that functional decline is accompanied by cortical shrinking and dopaminergic dry-out does not necessarily imply that the latter is causing the former, it may just as well be that both functional and biological decline is but the expression of the under-use of one’s cognitive potential. For the sake of the argument, we will leave this attractively radical possibility aside, however, and concentrate on our main point: that control needs active maintenance and that elderly may not exploit their full potential to engage therein.

According to a pessimistic interpretation of our reasoning one may be tempted to leave the basic rationale of current aging research intact and simply add the lack of maintenance to the list of dependent measures. According to this view, biological aging leads to both a reduction of cognitive resources and the loss of the ability to efficiently cope with this reduction. But a more optimistic interpretation is also possible. Even if one buys into the assumption that immutable biological factors cause reductions of cognitive-control resources, one could still seek to support the elderly individual’s ability to deal with, and at least partially compensate for such reductions. Attempts to do so may raise ethical issues. Note that many, if not all failures to exploit one’s potential to practice cognitive control arguably reflect individual preferences: people who feel vulnerable simply do not like to be exposed to situations that they think might reveal these vulnerabilities. Would it be socially responsible and ethically acceptable to encourage elderly with reduced cognitive resources to actively seek cognitively challenging, demanding situations? Even if doing so would reduce the challenge on the long run and turn the downward spiral into an upward spiral, encouraging needy individuals to take this step raises a number of issues that, we do not deny.

And yet, we strongly believe that a more serious consideration of the interdependence between cognitive control and physical and social interactions in theory, experimental practice, and societal reality is badly needed. With respect to research, we need a more systematic investigation and understanding of how the use of cognitive control processes affects their availability and efficiency. With respect to societal conditions, we need to ask whether policy provides sufficient support for self-empowerment of aging individuals. From an embodiment perspective, the probably most effective strike against human cognitive control abilities are retirement laws. In fact, retirement systematically undermines possible efforts to maintain the control abilities under biological challenge by eliminating various (job-related) sources of uncertainty and surprise, cutting substantial parts of the social network, at least for a substantial part of the day, and by possibly taking away opportunities for physical practice (depending on the job). Indeed, there is ample evidence that retirement is specifically associated with a general loss of processing speed (de Grip et al., 2015) and general cognitive functioning (Bonsang et al., 2012). Retirement selectively increases the rate of decline of cognitive abilities, presumably due to a lack of motivation to invest in compensatory activities (Mazzonna and Peracchi, 2012). From our point of view, a responsible societal response to biological aging must not prevent elderly individuals from practicing their cognitive-control skills (as present retirement laws do) but, rather, provide stronger individual and social reinforcement and more opportunities for elderly to engage in control-demanding activities, including physical and social interactions with substantial degrees of uncertainty and surprise.

## Author Contributions

All authors listed have made substantial, direct and intellectual contribution to the work and approved it for publication.

## Conflict of Interest Statement

The authors declare that the research was conducted in the absence of any commercial or financial relationships that could be construed as a potential conflict of interest.
